# Winner of the Analytical Science Advances Young Scientist Award 2022 at the 25th Norwegian symposium on chromatography: Christine Olsen

**DOI:** 10.1002/ansa.202300040

**Published:** 2023-07-29

**Authors:** Christine Olsen, Sebastiaan Eeltink

**Affiliations:** ^1^ Department of Chemistry University of Oslo Oslo Norway; ^2^ Hybrid Technology Hub ‐ Centre of Excellence University of Oslo Oslo Norway; ^3^ Department of Chemical Engineering Vrije Universiteit Brussel Brussels Belgium

The 25^th^ Norwegian Symposium on Chromatography took place in September 2022 in Sandefjord, Norway. This conference was attended by approximately 200 participants from various sectors, including industry, hospitals, and academia. One of the parallel oral sessions organized was specifically dedicated to emerging PhD researchers and post‐doctoral fellows. It was a pleasure to witness the exceptional quality of presentations and the enthusiasm displayed by the presenters. Consequently, the task of the jury, composed of Dr. Åse Marit Leere Øiestad from the Department of Forensic Sciences at Oslo University Hospital, Associate Prof. Cato Brede from the Department of Medical Biochemistry at Stavanger University Hospital, and Prof. Sebastiaan Eeltink from the Department of Chemical Engineering at Vrije Universiteit Brussel and Editor‐in‐Chief of Analytical Science Advances, was indeed challenging as they undertook the responsibility of selecting the best young scientist. After careful deliberation, Christine Olsen (Fig. 1) was chosen as the recipient of the award for an exceptional lecture addressing the key challenges and solutions to obtaining a sensitive and reliable determination of insulin secretion in stem cell‐derived islets using conventional liquid chromatography (LC) with triple quadrupole mass spectrometry (MS). Interestingly, this was her first “live” presentation outside of the university following the coronavirus disease 2019 pandemic and zoom‐conferences. Below is an interview with the recipient, where Analytical Science Advanced asked Christine Olsen questions about her PhD research as well as her general interests and hobbies.

**FIGURE 1:**
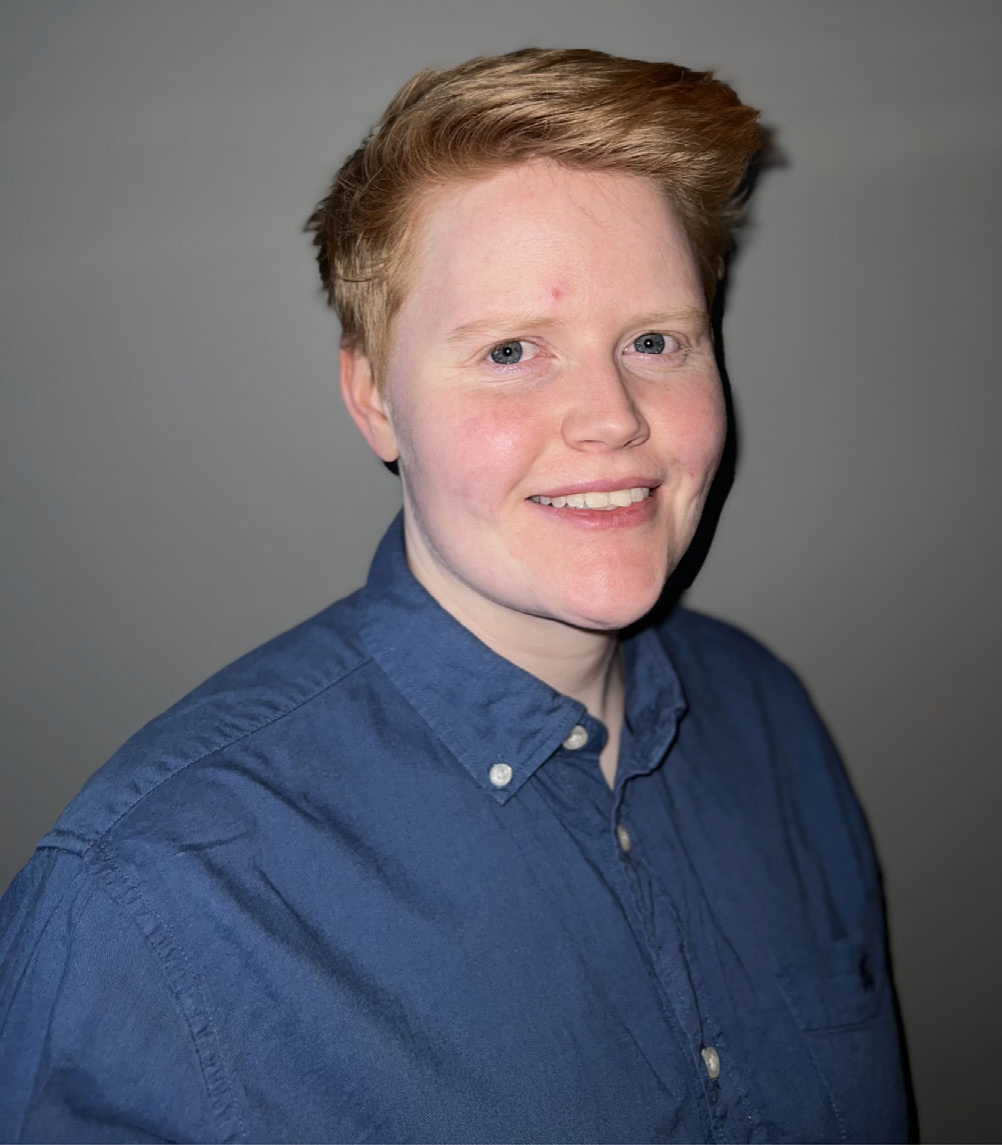
Christine Olsen: winner of the Analytical Science Advanced Young Scientist Award 2022.

## 1 CAN YOU BRIEFLY EXPLAIN THE TOPIC OF YOUR PHD RESEARCH?

**FIGURE 2:**
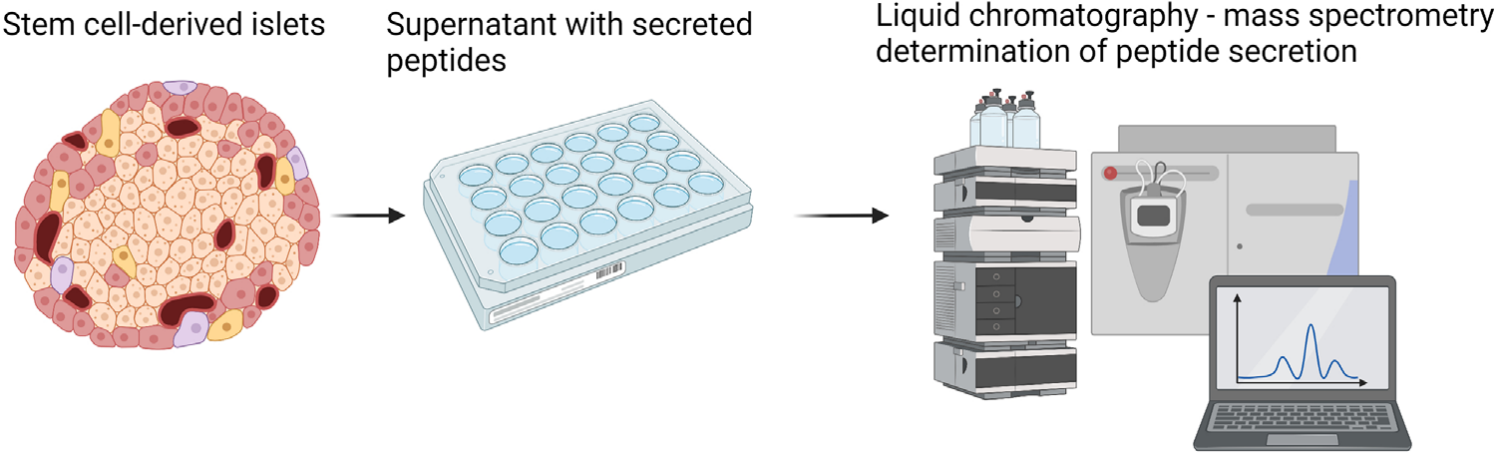
To examine secreted peptides in supernatant collected from stem cell‐derived islets, we apply liquid chromatography to obtain separation of our peptides of interest from other components in the sample. Subsequently, mass spectrometry is applied to detect the components eluted from the liquid chromatography system. By combining liquid chromatography and mass spectrometry we obtain a highly selective and sensitive method for the determination of secreted peptides.

My PhD research has primarily focused on developing a LC‐MS method for the determination of glucose regulatory peptides. The main objective of our study is to characterize the production and secretion of insulin, somatostatin‐14, and glucagon from stem cell‐derived islets. This collaborative effort involves the Hybrid Technology Hub Center of Excellence at the University of Oslo and the Department of Transplantation Medicine at Oslo University Hospital. The combined research is aimed at gaining a deeper understanding of human islet cell biology and advancing the development of beta cell replacement therapy for type 1 diabetes, see Figure 2 for the workflow. The differentiation of human stem cells into mature insulin‐producing islets may hold the potential to become an unlimited source of donor materials for patients with type 1 diabetes. As such, the characterization using highly specific LC‐MS has been instrumental in contributing to this critically important research.

The take‐home message from my lecture presented at the Norwegian Symposium on Chromatography was to highlight the significant impact of the non‐defined adsorption of insulin when utilizing different tubing configurations in an LC‐MS setup. The aim was also to emphasize the transformative possibilities that arise from eliminating this adsorption phenomenon. In the initial experiment conducted with insulin on the applied set‐up, we examined a new guard cartridge applying a phenyl/hexyl stationary phase. We observed that the insulin peak area was increasing for the three first injections, followed by extreme variation in the peak area in the following injections. These findings and combined with our previous efforts into obtaining a method for insulin analysis, we were able to discern that the peak area variation was due to non‐defined adsorption on the tubing.

## 2 WHY DID YOU CHOOSE A PATH INTO SCIENTIFIC RESEARCH?

After high school I got accepted into medical school, however I quickly discovered that I was too restless to be a physician. I probably would not have provided the patients with sufficient care! Having always had a drive to help others and to obtain more knowledge, I shifted into natural sciences and found my place in the laboratory trying to solve how much caffeine there was in a bag of tea using an old liquid chromatography pump combined with ultraviolet detection. From there, the ball kept rolling from a bachelor project, into a master thesis, and now getting to the end of my time as a PhD student.

## 3 WHO WERE THE MOST INFLUENTIAL PEOPLE IN YOUR PATH AS A SCIENTIST?

There were three key influential people shaping my way from a bachelor student in chemistry to a scientist. First, my supervisor Dr. Ole Kristian Brandtzæg on my first analytical course at the university took me under his wing and guided me into a bachelor project in his research group. The second was the group leader, Prof. Elsa Lundanes with her outstanding never‐ending source of knowledge being shared with all of the students. Additionally, I must express my gratitude to my current supervisor, Prof. Steven Ray Wilson. Under his mentorship, I have come to realize the immense significance and wide‐ranging applications of analytical chemistry. Moreover, he has been a constant source of great support throughout my PhD.

## 4 WHAT DO YOU CONSIDER TO BE THE MORE EXCITING TOPICS IN YOUR FIELD?

I believe that the rapid development of instrumental technology being done in both chromatography and MS is leading to endless possibilities moving the field forward. Personally, what excites me the most about analytical chemistry is its potential contribution in domains, such as clinical settings and pharmaceutical development. Moreover, the impact of stem cell technology and the development of organoids, which are 3D laboratory‐grown organ models, holds great promise for the future. These innovative models have the potential to serve as alternatives to animal models in drug development, and witnessing their impact will be truly inspiring.

## 5 WHAT WILL YOU DO AFTER YOUR PHD STUDY?

Having been at the University of Oslo for over 11 years it feels like it's time to experience the world outside of academia and I am aiming for a position connected to research and/or development with an output to help others. However, I would not be surprised if I eventually come back to academia, as being a PhD student in the bioanalytic research group at the Department of Chemistry at the University of Oslo has been extremely challenging, but also equally gratifying. I am forever grateful to the people I have met and the collaborations we have had. The motivation for my PhD was even more increased by being able to contribute (to a degree) to patients with diabetes, which resonated with my first idea of becoming a physician, and I hope to contribute more going forward.

## 6 WHAT NONSCIENTIST INSPIRES YOU THE MOST AND WHY?

Outside of science, I like to spend time with my family and people that matter. But, you would most likely find me on the floor at home cuddling with my pet bunnies, or at a concert venue enjoying good music. I am inspired by people with a natural born talent for what they can achieve, but also by the regular person on the street doing something good for others or the hard‐working people overcoming the challenges given to them by life.

